# The Neuroprotective Effects of Muscle-Derived Stem Cells via Brain-Derived Neurotrophic Factor in Spinal Cord Injury Model

**DOI:** 10.1155/2017/1972608

**Published:** 2017-07-05

**Authors:** Donghe Han, Shurui Chen, Shiqiang Fang, Shiqiong Liu, Meihua Jin, Zhanpeng Guo, Yajiang Yuan, Yansong Wang, Chang Liu, Xifan Mei

**Affiliations:** ^1^Department of Neurobiology, Jinzhou Medical University, Jinzhou, Liaoning Province 121000, China; ^2^Department of Orthopedics, The First Affiliated Hospital of Jinzhou Medical University, Jinzhou, Liaoning Province 121000, China; ^3^Department of Immunology, Jinzhou Medical University, Jinzhou, Liaoning Province 121000, China; ^4^Department of Endocrinology, The First Affiliated Hospital of Jinzhou Medical University, Jinzhou, Liaoning Province 121000, China

## Abstract

Muscle-derived stem cells (MDSCs) possess multipotent differentiation and self-renewal capacities; however, the effects and mechanism in neuron injury remain unclear. The aim of this study was to investigate the effects of MDSCs on neuron secondary injury, oxidative stress-induced apoptosis. An in vivo study showed the Basso, Beattie, and Bresnahan (BBB) score and number of neurons significantly increased after MDSCs' transplantation in spinal cord injury (SCI) rats. An in vitro study demonstrated that MDSCs attenuated neuron apoptosis, and the expression of antioxidants was upregulated as well as the ratio of Bcl-2 and Bax in the MNT (MDSCs cocultured with injured neurons) group compared with the NT (injured neurons) group. Both LC3II/LC3I and *β*-catenin were enhanced in the MNT group, while XAV939 (a *β*-catenin inhibitor) decreased the expression of nuclear erythroid-related factor 2 (Nrf2) and LC3II/LC3I. Moreover, MDSCs became NSE- (neuron-specific enolase-) positive neuron-like cells with brain-derived neurotrophic factor (BDNF) treatment. The correlation analysis indicated that there was a significant relation between the level of BDNF and neuron injury. These findings suggest that MDSCs may protect the spinal cord from injury by inhibiting apoptosis and replacing injured neurons, and the increased BDNF and *β*-catenin could contribute to MDSCs' effects.

## 1. Introduction

Spinal cord injury (SCI) is one of the most devastating, traumatic conditions, accompanied by initial mechanical damage and secondary injuries [1]. The mechanical damage of nerve axons by a primary injury induces biochemical and cellular cascades. Therefore, transplanting stem cells is considered the most effective method to treat SCIs [2], and various types of cells have been used to regenerate and replace the injured neurons to improve functioning [3–5]. Despite ongoing efforts, stem cell grafting has not achieved the desired effects because of transplant rejection and donor cell demise [6]. Recently, accumulating data have suggested that secondary injures, such as oxidative stress and apoptosis, could impede the successful treatment of SCIs by causing irreversible damage to nerve tissues. In addition, the poor survivability of transplanted cells is due to secondary injury processes [7]. Combined with the fact that the incidence of traumatic spinal cord injury is increasing worldwide [8], this has prompted the search for more effective novel cells.

Muscle-derived stem cells (MDSCs) are a multipotent, somatic stem cell that can be easily obtained from skeletal muscle. MDSCs can be transplanted as an autologous graft and can be rapidly expanded in vitro to reach the clinically relevant numbers of cells. They are also advantageous because they are much more resistant to a toxic microenvironment such as hypoxia, attenuate fibrosis, and readily differentiate into muscle, bone, neural, endothelial, fibroblastic, and hematopoietic lineages [9, 10]. Most importantly, MDSCs have an extended replicative lifetime [11]. However, the effects of MDSCs in neuron regeneration and proliferation are largely unknown. In the present study, we used SCI rats and primary cultured neurons to investigate the effects of MDSCs on neuron injury after SCI.

## 2. Materials and Methods

### 2.1. SCI Model, Cell Transplantation, and Behavioral Test

**Figure 1:**
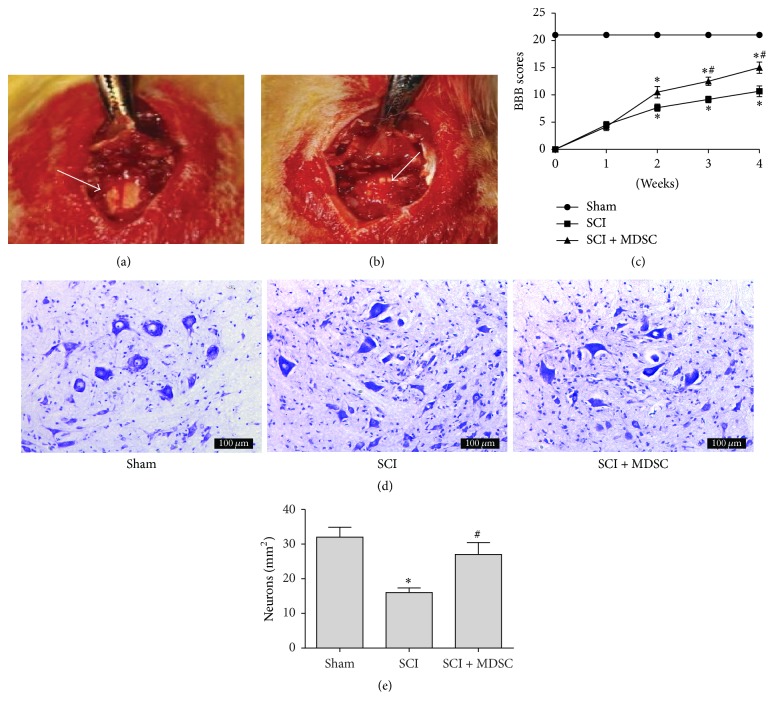
*Functional recovery after spinal cord injury*: (a) exposed spinal cord (arrow shows intact spinal cord); (b) transected edges of spinal cord (arrow); (c) locomotor function was assessed by BBB test after spinal cord injury (SCI). (d) Nissl stain was performed 4 weeks after SCI. (e) Quantification of surviving neurons. Data was shown as mean ± SEM (*n* = 3); ^*∗*^*P* < 0.05 versus sham. ^#^*P* < 0.05 versus SCI. Scale bar: 100 *μ*m.

Male adult SD rats weighing 200–220 g were randomly divided into three groups of sham-operated (*n* = 6), SCI (*n* = 6), and SCI + MDSC (*n* = 6). Laminectomies were carried out under anesthesia at the T9-T10 level (Figures 1(a) and 1(b)). One week after spinal cord injury, MDSCs (approximately 5 × 10^5^ in 5 *μ*l saline) were injected in the rostral and caudal of the lesion using a Hamilton syringe. An immunosuppressant and antibiotics were administered 1 day before transplantation and every day throughout the experiments. The locomotor function was assessed by the Basso, Beattie, and Bresnahan (BBB) locomotor rating scale once a week for 4 weeks. Four weeks after SCI, spinal cords were used for Nissl staining.

### 2.2. MDSC and Neuron Isolation

Newborn Sprague-Dawley rats were obtained from the Laboratory Animal Centre of Jinzhou Medical University (License umber SYXK (Liao) 2003-0011). The skeletal muscles from hind limbs were subjected to the isolation of MDSCs by preplating methods [12]. Muscles were cut into pieces, digested with a mixed enzyme solution (2.4% type II dispersion enzyme, 1% type II collagenase, and 2.5 mmol/L CaCl2; Sigma, St. Louis, MO, USA) at 37°C for 45 min., and filtered through a stainless-steel mesh with a pore diameter of 200 *μ*m. The cells were centrifuged at 1,000 rpm for 10 min., and the pellets were resuspended in DMEM (Gibco, Santa Clara, CA, USA) with 10% fetal calf serum (Gibco), 10% horse serum, and 0.5% chick embryo extract (US Biological, Marblehead, MA, USA). Cells were cultured in 25 mL culture flasks for 2 hours (recorded as preplate1 or pP1) and then centrifuged at 1,000 rpm for 10 min., followed by daily transfers of nonadherent cells and replatings for 5 days until preplating 6 (pP5). All animal experiments were conducted in accordance with the National Institute of Health Guide for the Care and Use of Laboratory Animals.

The brain cortex was isolated from the same rats and gently dissociated to release the neurons. The cell suspensions were plated at a density of 1 × 10^6^/mL with a Neurobasal Medium (Gibco) supplemented with 1 mM glutamine (Amresco, OH, USA), 100 U/mL penicillin/streptomycin, and B27 (Gibco). The cells were grown in a humidified incubator maintained at 37°C with 5% CO_2_.

### 2.3. The Coculture System and Grouping

To mimic injured conditions of spinal cord injury in vitro, primary neurons isolated from the brain (NC) were cultured with tert-butyl hydroperoxide (t-BHP, 200 *μ*mol/L) (Sigma), for 4 h. Then the injured neurons (5 × 10^6^ cells/well) were cultivated without (NT group) or with (MNT group) MDSCs (1 × 10^6^ cells/well) or treated with XAV939 (8 *μ*M, MNT + XAV939) (MedChem Express, NJ, USA). The supernatant was used to measure BDNF, and injured neurons were subjected to staining and apoptosis.

To confirm the effects of brain-derived neurotrophic factor (BDNF) on the MDSCs, we set up an induced group in which MDSCs were cultured with 20 ng/mL BDNF (B3795, Sigma, USA) and an MDSC group without BDNF as a control. The morphological changes of the cells were observed under a phase-contrast microscope, and neuron-specific enolase (NSE) staining was performed after 6 days.

### 2.4. Nuclear Staining for Apoptotic Neurons

Cell climbing sheets on the second, fourth, and sixth days were fixed in 4% paraformaldehyde. After staining with Hoechst 33258 (5 *μ*g/mL; 94403, Sigma), the cells were observed under an inverted fluorescent microscope. Five fields were randomly chosen, and the apoptotic cells were counted.

### 2.5. Flow Cytometry Analysis for t-BHP-Induced Apoptosis

To quantify cell apoptosis, an annexin V-fluorescein isothiocyanate (V-FITC)/propidium iodide (PI) (sc-4252 AK, Santa Cruz Biotechnology, Santa Cruz, CA, USA) double staining assay was performed per the manufacturer's instructions.

### 2.6. RNA Extraction and qPCR

After 6 days' culture, neurons were collected, and total RNA was isolated using a TRIzol reagent; 5 *μ*g of each sample was used to produce cDNA. Reverse transcription and amplification of target genes were performed according to the manufacturer's instructions (11-752 and FERK0241, Invitrogen, CA, USA). The PCR reactions were carried out at 95°C for 2 min., followed by 40 cycles of 95°C for 15 s., 60°C for 30 s., and 72°C for 30 s. with a final extension at 72°C for 5 min. GAPDH was used as an internal control. The primer sequences are shown in Table 1.

### 2.7. Western Blot

After 6 days' culture, neurons were collected and homogenized in RIPA buffer (Tris-HCl, pH 7.4), 150 NaCl, 2 EDTA, 1% IGEPAL, 0.1% SDS, protease inhibitor cocktail, and phosphatase inhibitor. Subsequently, 30 *μ*g of protein was separated by PAGE gel and transferred to a PVDF membrane. After blocking in Tris buffer saline-1% nonfat dry milk for 1 h., membranes were probed with primary antibody Nrf2 (1 : 1,000, ab31163), LC3 (1 : 1,000, ab48394), and *β*-catenin (1 : 1,000, ab32572) overnight at 4°C. HRP-conjugated secondary antibodies were used for the detection of primary antibodies (1 : 10,000, ab6721). GAPDH (1 : 2,000, ab9485) was used as a control in the same sample. Signals were visualized using the ECL kit (34080, Thermo Fisher Scientific, USA). A densitometry analysis was conducted using ImageJ software (NIH, Bethesda, MD). All experiments were repeated at least 3 times (*n* = 3) using independent samples. All antibodies were purchased from Abcam (Abcam, Cambridge, MA, USA).

### 2.8. BDNF-Release Assay

To measure BDNF levels, the cell-cultured supernatants (Day 2, Day 4, and Day 6) were subjected to an enzyme-linked immunosorbent assay (EK0307-RB, Wuhan Boster, China). The assay was performed according to the manufacturer's protocol.

### 2.9. Immunocytochemistry

The MDSCs were fixed with an ice-cold 4% paraformaldehyde, incubated with 3% H2O2 for 30 min., and blocked with goat serum. The cells were then incubated with neuron-specific enolase (NSE) (1 : 100, sc-51880) overnight at 4°C, followed by a goat anti-mouse IgG (1 : 100; sc-2005, Santa Cruz Biotechnology, USA). Next, cells were incubated with DAB solution (D3939, Sigma, USA).

### 2.10. Statistical Analysis

The results are presented as the mean ± SEM. Analysis of variance (ANOVA) was performed to determine the differences among the groups. Post hoc tests were conducted using Bonferroni comparisons. A Pearson correlation analysis was performed to investigate the relation between the number of apoptotic cells and expression of BDNF.  Table 2 provides a good indication of the qualitative description of the strength of the linear relationship and the absolute value of *R*. A *P* value <0.05 was considered statistically significant.

## 3. Results

### 3.1. Effects of MDSC on Spinal Cord-Injured Rats

To examine the effects of MDSCs on spinal cord injury, locomotor function recovery after SCI was assessed with the BBB test, and there was no significant difference between the two SCI groups before MDSC transplantation. Prior to treatment, the mean BBB Locomotor Scores of all SCI rats were significantly decreased. In the first week posttreatment, a significantly faster functional recovery was observed in the SCI + MDSC group. The improvement in locomotor behavior was significant and could be observed until the end of the experiment (Figure 1(c)).

To determine the effects of MDSC on spinal cord tissue, a Nissl staining was performed. The number of surviving neurons was lower in the SCI group than in the sham group (*P* = 0.002); however, the number of surviving neurons increased more in the SCI + MDSC group than the SCI group (*P* = 0.034). These results demonstrate the significant protective effect of MDSCs (Figures 1(d) and 1(e)).

### 3.2. Effects of MDSCs on Neuron Apoptosis

**Figure 2:**
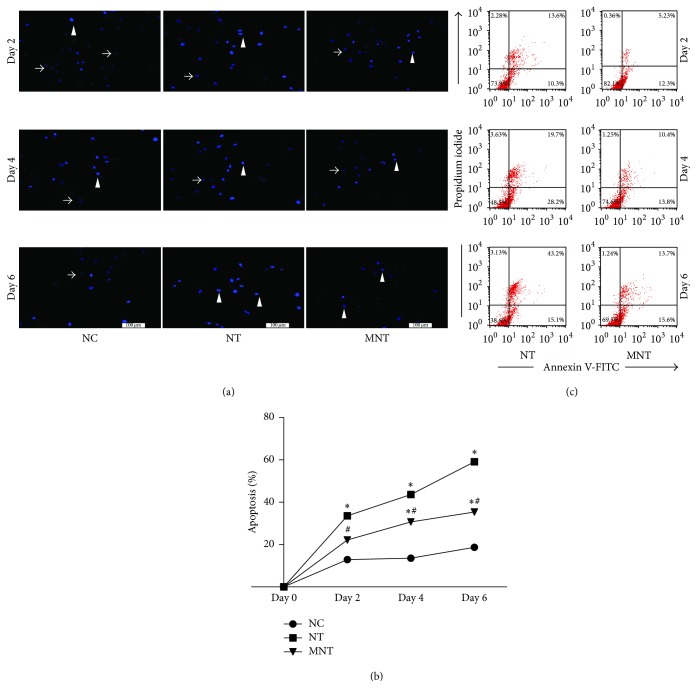
*Effects of MDSCs on neurons*. (a) The neurons stained with Hoechst 33258. Arrowheads (▲) indicate normal neurons and arrows (↑) indicate apoptotic neurons. Five fields were randomly selected and the (b) apoptotic rates were measured. (c) The ratio of apoptosis of neurons was measured by Annexin V-FITC/propidium iodide double staining. Data was shown as mean ± SEM (*n* = 5), ^*∗*^*P* < 0.05 versus NC group, and ^#^*P* < 0.05 versus NT group. NC (no treatment); NT (neurons treated with t-BHP); MNT (neurons cocultured with MDSCs after treatment with t-BHP). Scale bar: 100 *μ*m.

As shown in Figure 2, the ratio of cell apoptosis of the NT group gradually increased with time. The apoptosis rate of the MNT group also showed an increase; however, that of the MNT group was significantly lower than the NT group (Figures 2(a) and 2(b)).

The flow cytometric analysis showed that there were far fewer apoptotic cells in the MNT group than in the NT group at the same time point, although the number of apoptotic cells in the MNT group was still higher than in the NC group (Figure 2(c)).

### 3.3. Effects of MDSCs on Oxidative Stress-Induced Apoptosis

**Figure 3:**
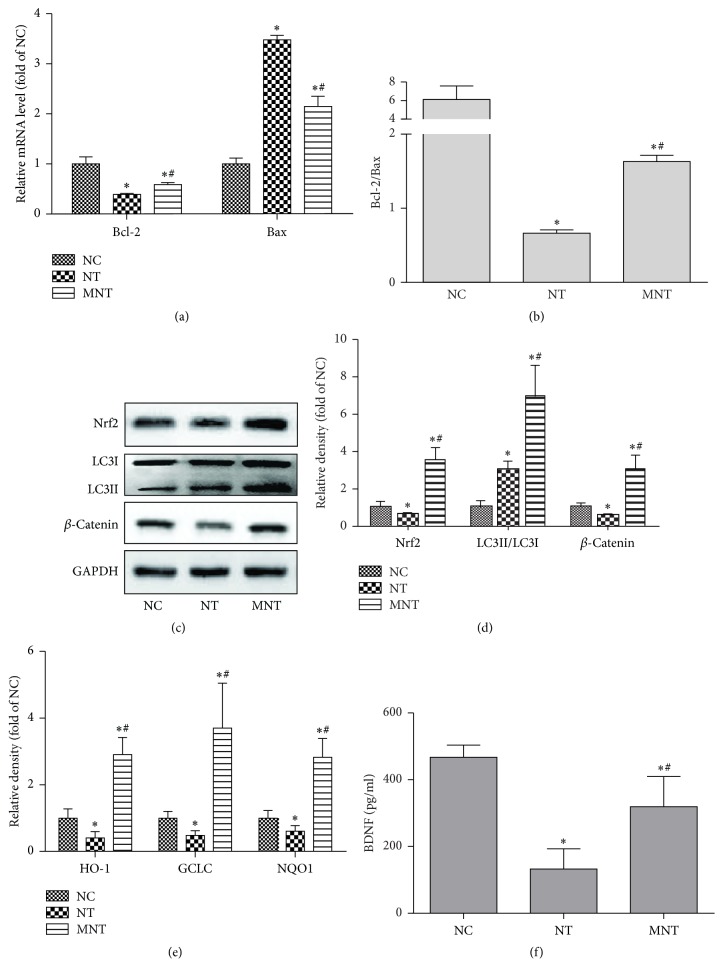
*Effects of MDSC on expression of apoptosis-related factors*. (a) Total RNA extracted from the cells was used for detecting the mRNA level of Bcl2 and Bax and (b) Bcl2/Bax ratio. (c) Total protein was used to observe the expression of Nrf2, LC3, and *β*-catenin and (d) the relative density. (e) The mRNA expression of antioxidants (*n* = 3). (f) BDNF in culture media (Day 6) was measured with ELISA kit (*n* = 6). Data was shown as mean ± SEM, ^*∗*^*P* < 0.05 versus NC group, and ^#^*P* < 0.05 versus NT group.

Compared to the NC group, the gene expression of Bcl-2 decreased in the NT group and MNT group (*P*_NT_ = 0.0001,  *P*_MNT_ = 0.0001), while the expressions of the Bax in both groups were increased (*P*_NT_ = 0.0001,  *P*_MNT_ = 0.0081). There was, however, more Bax gene expression in the NT group than in the MNT group (*P* = 0.0028), while the expression of Bcl-2 was less in the NT group than in the MNT group (*P* = 0.0079), and the ratio between Bcl-2 and Bax in the MNT group was higher than in the NT group (*P* = 0.0005) (Figures 3(a) and 3(b)).

The expression of an antioxidant, Nrf2, and its target genes, HO-1, GCLC, and NQO1, decreased in the NT group (*P*_Nrf2_ = 0.0154,  *P*_HO-1_ = 0.0126,  *P*_GCLC_ = 0.0187,  *P*_NQO-1_ = 0.0204), while those in the MNT group were enhanced compared with the NC group (*P*_Nrf2_ = 0.0001,  *P*_HO-1_ = 0.0001,  *P*_GCLC_ = 0.0001, and *P*_NQO1_ = 0.0033). The ratio of LC3II and LC3I was also increased in both NT and MNT groups (*P*_NT_ = 0.0015,  *P*_MNT_ = 0.0001). On the contrary, the levels of aforementioned proteins and genes in the MNT group were significantly high in comparison to the NT group (*P*_Nrf2_ = 0.0001,  *P*_HO-1_ = 0.0001,  *P*_GCLC_ = 0.0001,  *P*_NQO-1_ = 0.0001, and  *P*_LC3II/LC3I_ = 0.0001) (Figures 3(c)–3(e)).

### 3.4. Effects of MDSCs on BDNF

To investigate the effects of MDSCs on growth factors, the expression of BDNF in supernatant was examined. The level of BDNF at day 6 in the NT group and MNT group decreased relative to the NC group (*P*_NT_ = 0.0001,  *P*_MNT_ = 0.0122). However, the level of BDNF was much higher in the MNT group than in the NT group (*P* = 0.0024) (Figure 3(f)).

### 3.5. Effects of MDSCs on *β*-Catenin

**Figure 4:**
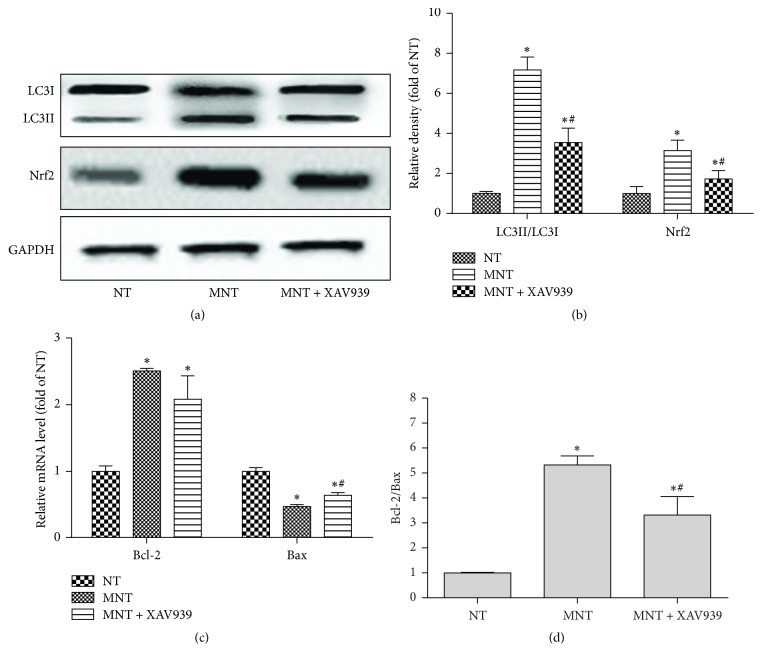
*Effects of XAV939 on neuron apoptosis*. To investigate the roles of *β*-catenin, XAV939 (*β*-catenin inhibitor) was added to MNT group. (a) Total protein was used to observe the expression of Nrf2 and LC3 and (b) the relative density. (c) Total RNA extracted from the cells was used for detecting the mRNA level of Bcl2 and Bax and (d) Bcl2/Bax ratio (*n* = 3). Data was shown as mean ± SEM, ^*∗*^*P* < 0.05 versus NT group, and ^#^*P* < 0.05 versus MNT group.

To investigate the mechanism of MDSCs, the expression of *β*-catenin was examined. As Figure 3 shows, *β*-catenin decreased in the NT group (*P* = 0.0401), while that in the MNT group increased compared with the NT group (*P* = 0.0001) (Figures 3(c) and 3(d)). In addition, increased LC3II/LC3I and Nrf2 in the MNT group were suppressed with XAV939 (*β*-catenin inhibitor) treatment (*P*_LC3II/LC3I_ = 0.0001,  *P*_Nrf2_ = 0.0001) (Figures 4(a) and 4(b)). Compared to the MNT group, the expression of Bax was upregulated, while the ratio of Bcl2/Bax was downregulated when adding XAV939 to the MNT group (*P*_Bax_ = 0.0309,  *P*_Bcl2/Bax_ = 0.028) (Figures 4(c) and 4(d)).

### 3.6. Morphological Changes of MDSCs

**Figure 5:**
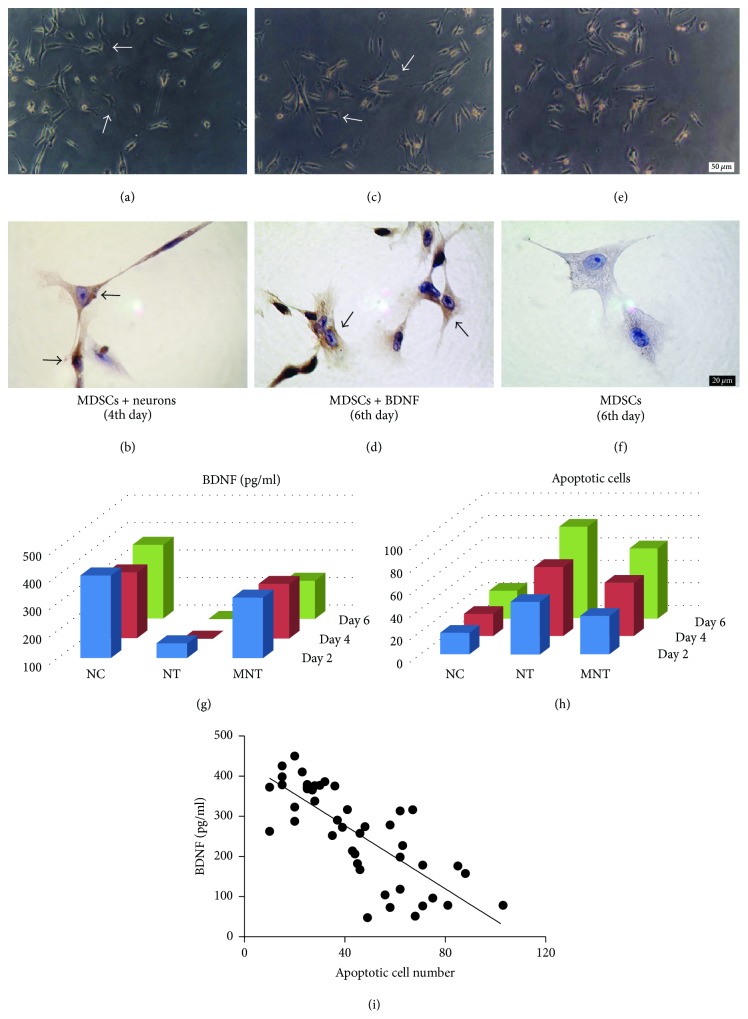
(a-b) The morphological changes of MDSCs in the MNT group. MDSCs were stained with NSE. (c–f) The effects of BDNF on MDSCs. MDSCs were cultured with (c, d) and without (e, f) BDNF for six days, and NSE staining was performed (d, f). Arrows (↑) indicate neuron-like cells (*n* = 3). Scale bar: (a, c, e) 50 *μ*m; (d, e, f) 20 *μ*m. (g) The level of BDNF in culture media was measured by ELISA (*n* = 6). (h) The number of apoptotic cells: after incubated with or without MDSCs, the neurons were stained with Hoechst 33258, and apoptotic cells were counted (*n* = 5). (i) The relationship between the number of apoptotic cells and the expression of BDNF. The linear regression analysis for in vitro study showed a negative correlation between BDNF and apoptosis (*R* = −0.775,  *P* < 0.0001).

To further verify the effects of BDNF on MDSCs' differentiation under the toxic microenvironment, we performed an in vitro study using primary neurons. The MDSCs of the MNT group showed morphological changes from the fourth day. The cells began to shrink and extend neurites (Figure 5(a)), which connected with one another as time progressed. To verify whether they had neuronal characteristics, the cells were stained with NSE, and it showed positive results for the MDSCs in the cocultured group (Figure 5(b)). In the experiment to test the effects of exogenous BDNF on the MDSCs, it is evident that the cytoskeleton retracted more progressively to the cell centre, and the extended neurites also connected with one another, which showed NSE-positive results (Figures 5(c) and 5(d)). The cells in the control group (MDSCs without BDNF) showed no morphological changes, and the NSE staining results were negative (Figures 5(e) and 5(f)).

### 3.7. The Relationship between Apoptosis and BDNF

The secretion of BDNF in the NT and MNT groups was gradually decreased from day 2, while the levels of BDNF in MNT groups were much higher than those in NT groups at the same time point (*P* < 0.001) (Figure 5(g)). Numbers of apoptotic cells in both coculture groups were gradually upregulated compared to the NC group. However, those in the MNT group were much lower compared with the NT group (*P* < 0.001) (Figure 5(h)).

To investigate the correlation between the number of apoptotic cells and levels of BDNF, a linear regression analysis was performed. Results showed a strong negative correlation regarding the number of apoptotic cells and the expression of BDNF (*R* = −0.775, *P* < 0.0001, Figure 5(i)), suggesting the possibility that decreasing BDNF contributes to the injury of neurons.

## 4. Discussion 

### 4.1. MDSCs Suppress the Progression of Apoptosis by Inhibiting Oxidative Stress and Inducing Autophagy

The secondary injury following spinal cord injury aggravates the primary lesion and exacerbates neurological impairment. Apoptosis is one of the major events in secondary injury, which affects the survivability of neurons and implanted cells [13]. During apoptosis progression, Bax is considered an important proapoptotic protein, and Bcl-2 prevents apoptosis by downregulating Bax [14]. Furthermore, accumulating data are showing that Nrf2 is the crucial regulator of apoptosis by activating antioxidant cascades, such as HO-1, GCLC, and NQO1 [15, 16], which contribute to the suppression of apoptosis. Our data presented here support the previous reports regarding the upregulation of apoptosis (Figure 2), and we also found that MDSCs could inhibit apoptosis via the upregulation of the ratio between Bcl-2 and Bax as well as antioxidants in part. Therefore, the data suggest that the protective effects of MDSCs on neuronal apoptosis, at least in part, result from the regulation of oxidative stress by upregulating Nrf2 and its downstream antioxidants (Figure 3).

Autophagy is another essential mechanism for maintaining cellular homeostasis under stress conditions. Many studies have reported that the activation of autophagy protects against pathologies [17, 18], and the inhibition of autophagy is associated with an increase of apoptosis, which may aggravate neuronal damage [19]. In the present study, we observed that MDSCs could promote the autophagy activation and suppress apoptosis in the MNT group compared with the NT group. Unexpectedly, we also observed the strong activation of autophagy and apoptosis in the NT group compared with the normal group (Figure 3). Yu et al. [20] have reported that autophagy is initiated within hours to protect neurons by disposing injured components, and after few days, autophagy could induce normal cell death in the SCI model. Since the apoptosis is suppressed by MDSCs, it suggests that increased autophagy in the NT group is associated with neuron death. However, MDSCs could extend the protective effect of autophagy.

### 4.2. Protective Effects of BDNF Secreted by MDSCs Are Associated with *β*-Catenin

Recently, many studies have focused on the effects of various neurotrophic factors secreted by stem cells [21] since many scientists have proposed that the terminal differentiation of other stem cells is not a major determinant of successful tissue and function repair [22, 23]. Brain-derived neurotrophic factor (BDNF), one of the major neurotrophic factors, regulates neuronal differentiation and proliferation [24]. Some studies have provided evidence that BDNF could suppress apoptosis via the upregulating ratio of Bcl-2 and Bax in different tissues [25, 26], and more recently, other reports have indicated that BDNF could upregulate Nrf2 [27] and autophagy [28]. The present study showed MDSCs promoted the secretion of BDNF (Figures 3 and 5), and the lower expression of BDNF was closely linked with the severity of injury (Figure 5(i)). Therefore, it is believed that MDSCs could protect the neurons from oxidative stress by BDNF.

Wnt/*β*-catenin signaling defines organizing centres that orchestrate neural development, axonal guidance, cell proliferation, and neural cell survival [29]. However, the roles of *β*-catenin in spinal cord injury are still controversial. Several studies have reported that *β*-catenin is activated after SCI for promoting functional recovery [30], and other studies have shown decreased *β*-catenin in SCI [31]. The different results might be related to the different time point after SCI because SCI includes complex physiological and biochemical mechanisms. Various proteins are increased immediately after SCI to compensate for the injury and then decreased with the passage of time [32]. Our results also showed that MDSCs could promote *β*-catenin, which is downregulated in injured neurons. The inhibition of *β*-catenin results in reduced autophagy, antioxidants, and increased apoptosis. The results are consistent with previous reports that indicated the roles of *β*-catenin on apoptosis and autophagy. It is reported that *β*-catenin could inhibit apoptosis via regulating CYP21A2 [33], and the alteration of *β*-catenin is closely associated with autophagy in SCI rats [31]. Interestingly, BDNF has been reported to regulate *β*-catenin [34]. Therefore, protective effects of MDSCs on injured neurons, at least in part, result from the increased presence of *β*-catenin, which is regulated by BDNF.

### 4.3. BDNF Could Stimulate Neuron-Like Cell Differentiation

In an experiment to further verify the roles of MDSCs under a stress microenvironment, we observed a large number of NSE-positive neuron-like cells in the MNT group (Figures 4(a)–4(d)). We also found that the presence of exogenous BDNF stimulated the transformation of MDSCs into NSE-neuron-like cells. Increasing studies have revealed that various stem cells also can be induced into neuron-like cells by treatment with BDNF [35, 36]. Hence, we speculate that MDSCs may primarily stimulate neurons to secrete BDNF, which could eventually affect the terminal differentiation of MDSCs into neuron-like cells. Neuron-like cells are also believed to replace injured neurons and exert neuronal-like roles under oxidative conditions. However, further studies need to investigate the exact roles of neuron-like cells in SCI rats.

## 5. Conclusions

Our findings showed that the decreased apoptosis rate by the MDSCs is related to the increased BDNF. BDNF might not only upregulate autophagy but also downregulate oxidative stress. Moreover, BDNF promotes the transition of MDSCs into neuron-like cells, which could replace injured neurons. In the present study, we also found *β*-catenin is involved with the protective effects of MDSCs by inducing antioxidants and autophagy. These results suggest that MDSCs could be potential therapeutic approaches for the treatment of spinal cord injuries.

## Figures and Tables

**Table 1:** Primers used for the gene expression analysis.

Genes^a^	Sequences^b^ (5′–3′)
Bcl-2	F: CTGGTGGACAACATCGCTCTG
	R: GGTCTGCTGACCTCACTTGTG
Bax	F: GCGAATTGGAGATGAACTGG
	R: GTGAGCGAGGCGGTGAGGAC
HO-1	F: GCCTGCTAGCCTG GTTCAAG
	R: AGCGGTGTCTGGGATGAACTA
GCLC	F: GTCCTCAGGTGACATTCCAAGC
	R: TGTTCTTCAGGGGCTCCAGTC
NQO1	F: GGCAGAAGAGCACTGATCGTA
	R: TGATGGGATTGAAGTTCATGGC
GAPDH	F: AAGCTGGTCTCAACGGGAAAC
	R: GAAGACGCCAGTAGACTCCACG

Bcl-2: B-cell lymphoma 2; Bax: Bcl-2-like protein 4; Nrf2: nuclear erythroid-related factor 2; HO-1: heme oxygenase1; GCLC: glutamate-cysteine ligase catalytic subunit; NQO1: NAD(P) H: quinine oxidoreductase-1.

**Table 2:** Strength of the correlation.

Absolute value of *R*	Qualitative description of the strength
0.8–1	Very strong
0.6–0.79	Strong
0.40–0.59	Moderate
0.2–0.39	Week
0–0.19	Very week
